# Experimental data used to validate the FE model of the structural performance of two flexible pipes laid in a single trench

**DOI:** 10.1016/j.dib.2019.104594

**Published:** 2019-10-01

**Authors:** Alaa Abbas, Felicite Ruddock, Rafid Alkhaddar, Glynn Rothwell, Iacopo Carnacina, Robert Andoh

**Affiliations:** a Department of Civil Engineering, Liverpool John Moores University, Henry Cotton Building, 15-21 Webster Street, Liverpool, L3 2ET, UK; b Department of Civil Engineering, Liverpool John Moores University, Peter Jost Centre, Byrom Street, Liverpool L3 3AF, UK; c Department of Maritime and Mechanical Engineering, Liverpool John Moores University, Byrom Street, Liverpool, L3 3AF, UK; d CEO and President of AWD Consult Inc., 32 Vista Drive, South Portland, ME 04106, USA

**Keywords:** Experimental data, FE model of buried pipes, Novel configuration, Sewer system, Soil-pipe interaction

## Abstract

The objective of the article is to describe the methodology followed to validate the finite element model for the new method of setting pipes in a separate sewer system, using one trench to accommodate the storm pipe over the sanitary pipe “doi.org/10.1016/j.tust.2019.103019” (Abbas et al., 2019). A physical model was established in the Liverpool John Moores University (LJMU) lab to test the structural performance of two PVC pipes buried in one trench. The results of the physical model were used to validate an FE model using the same material properties and boundary conditions used in the physical model. The validation process allowed the FE model to be upgraded to a 3D FE full-scale model for testing the novel method used to place the separate sewer system.

**Table:** Specifications Table

Subject	Civil Engineering
Specific subject area	Buried pipes
Type of data	Image (FE model, SolidWorks) Graph Figure
How data was acquired	Linear vertical displacement transducers (LVDTs, Micro-Measurements HS 50), GFRA-3-70 strain gauges and P3 Strain Indicator and Recorder
Data format	Raw and analysed
Parameters for data collection	The data was collected from experimental works, using a physical model in Liverpool John Moores University laboratory
Description of data collection	A physical model with dimensions of 2.5 × 0.5 × 1 m^3^ was built in the laboratory to test the performances of two PVC pipes. The physical model was embedded in a hydraulic rig used to provide lateral support for the trench walls and to apply the traffic load
Data source location	Liverpool John Moores University, UK
Data accessibility	Data is available in the article
Related research article	Alaa Abbas, Felicite Ruddock, Rafid Alkhaddar, Glynn Rothwell, Iacopo Carnacina and Robert Andoh Investigation of the Structural Performance of Two Flexible Pipes Set in One Trench with a New Placement Method for Separate Sewer Systems Tunnelling and Underground Space Technology https://doi.org/10.1016/j.tust.2019.103019

**Table:** 

**Value of the data** • Identifying the elasto-plastic soil properties to calculate the modified Drucker–Prager cap constitutive model parameters. • The data provided the experimental results for testing the two buried pipes lying in one trench, one over the other, under traffic load. • The data was used to validate the finite element model for the buried pipes, which can be used to test other different scenarios of positions for pipes buried in a trench. • The data presents the behaviour of the soil surface exposed to a traffic load when buried pipes are set underneath in different positions.

## 1 Data

**Fig. 1:**
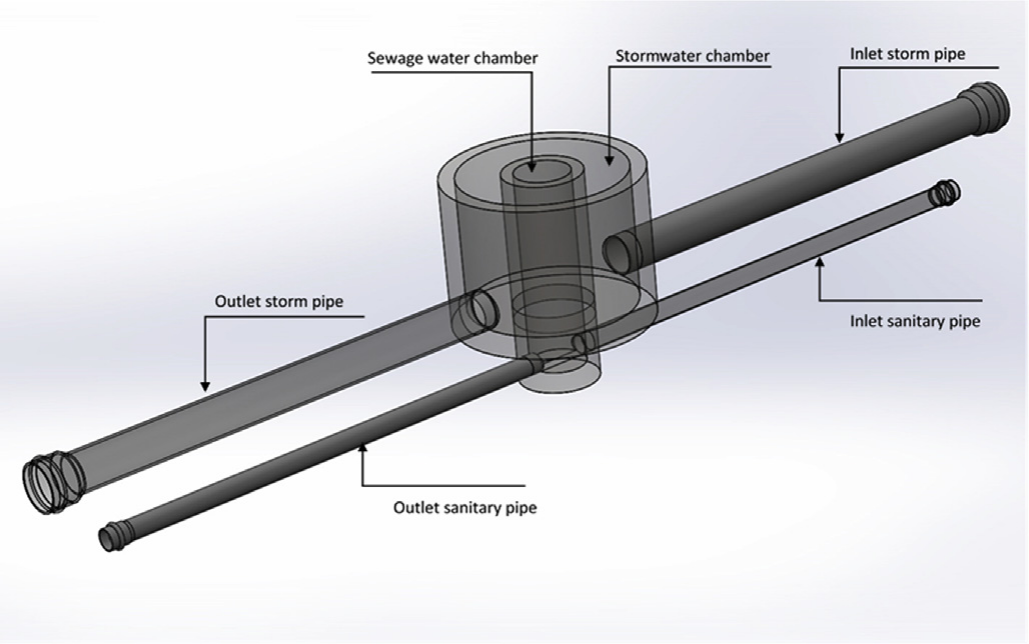
3D design of the innovative manhole [2].

**Fig. 2:**
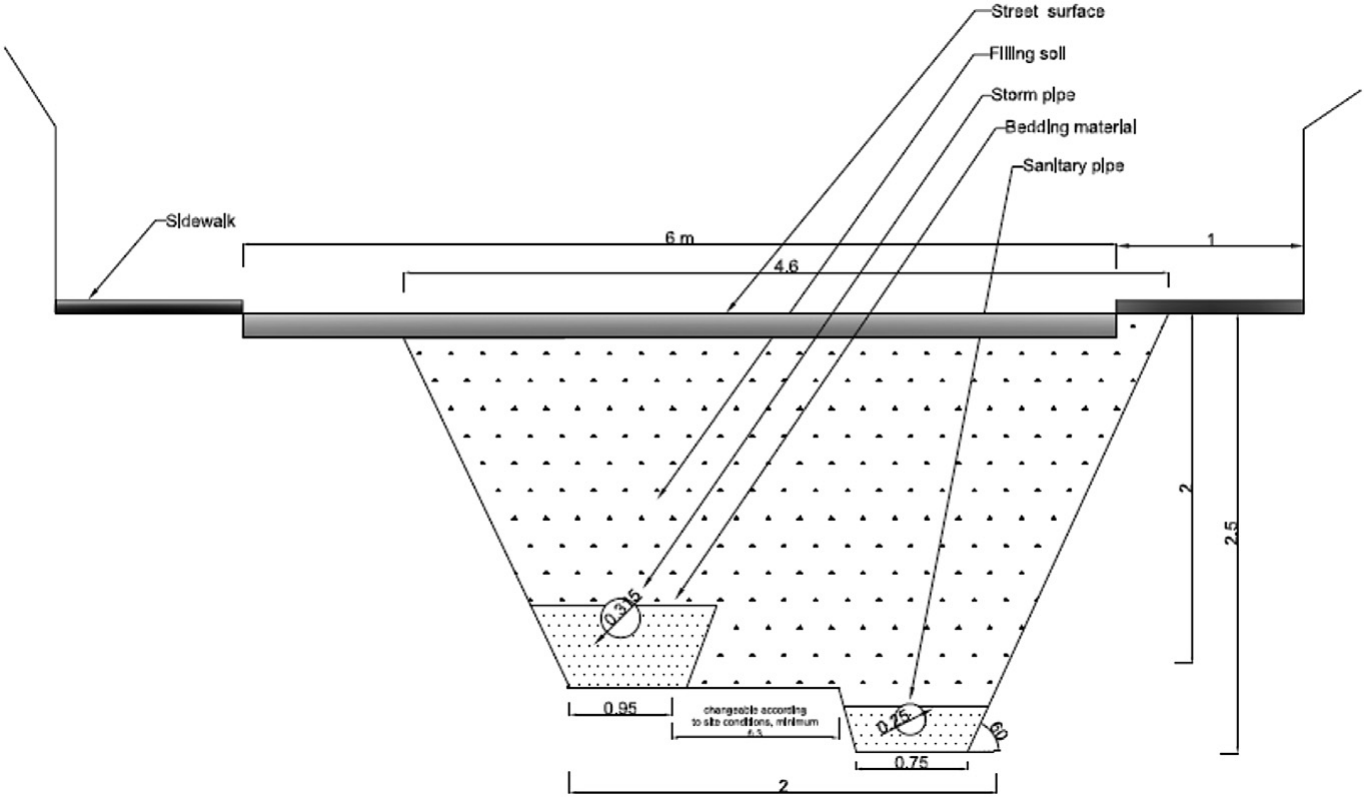
Conventional method for laying two pipes in a separate sewer system [3].

**Fig. 3:**
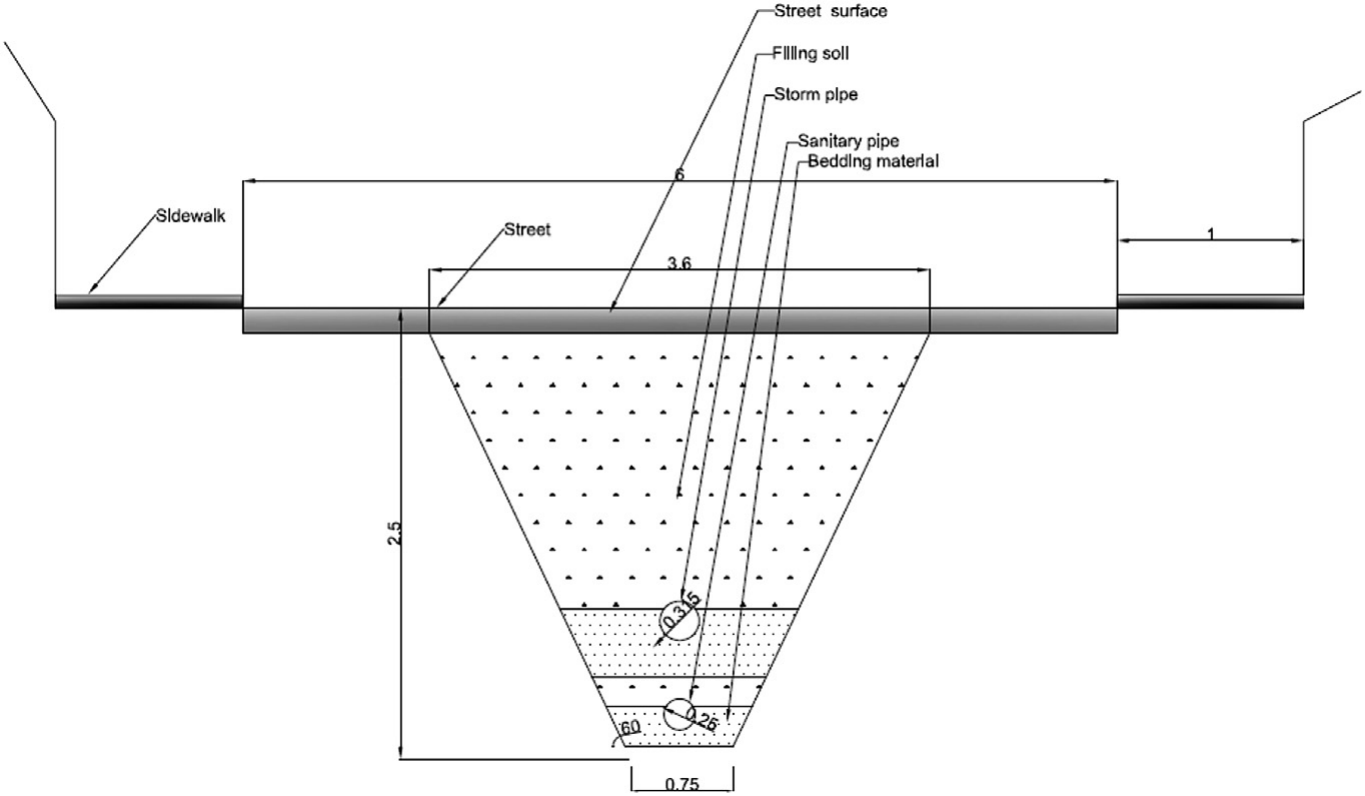
Innovative method of laying two pipes in a separate sewer system [3].

**Fig. 4:**
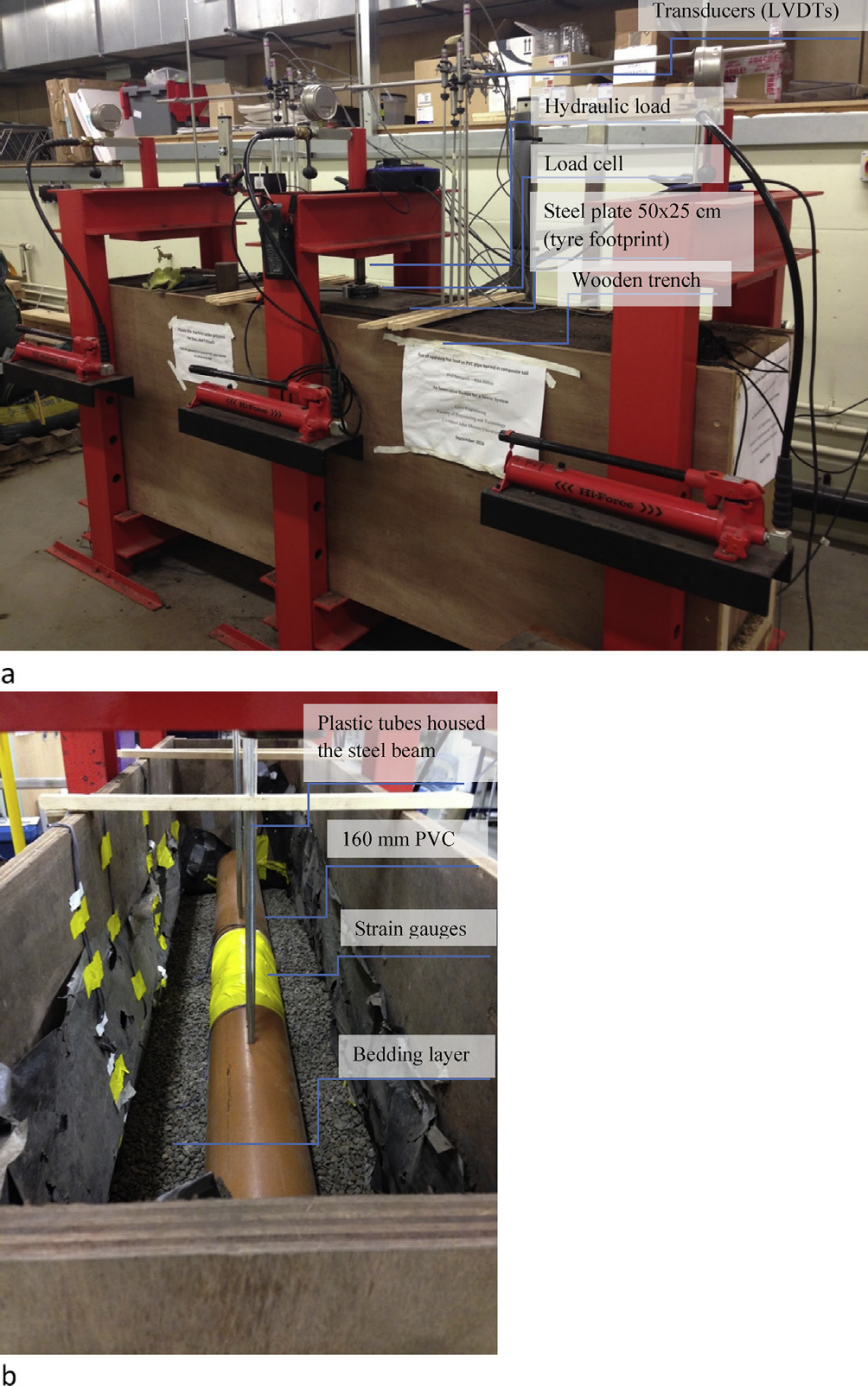
Configuration of the physical model in the laboratory equipped with measurement and recording devices.

**Fig. 5:**
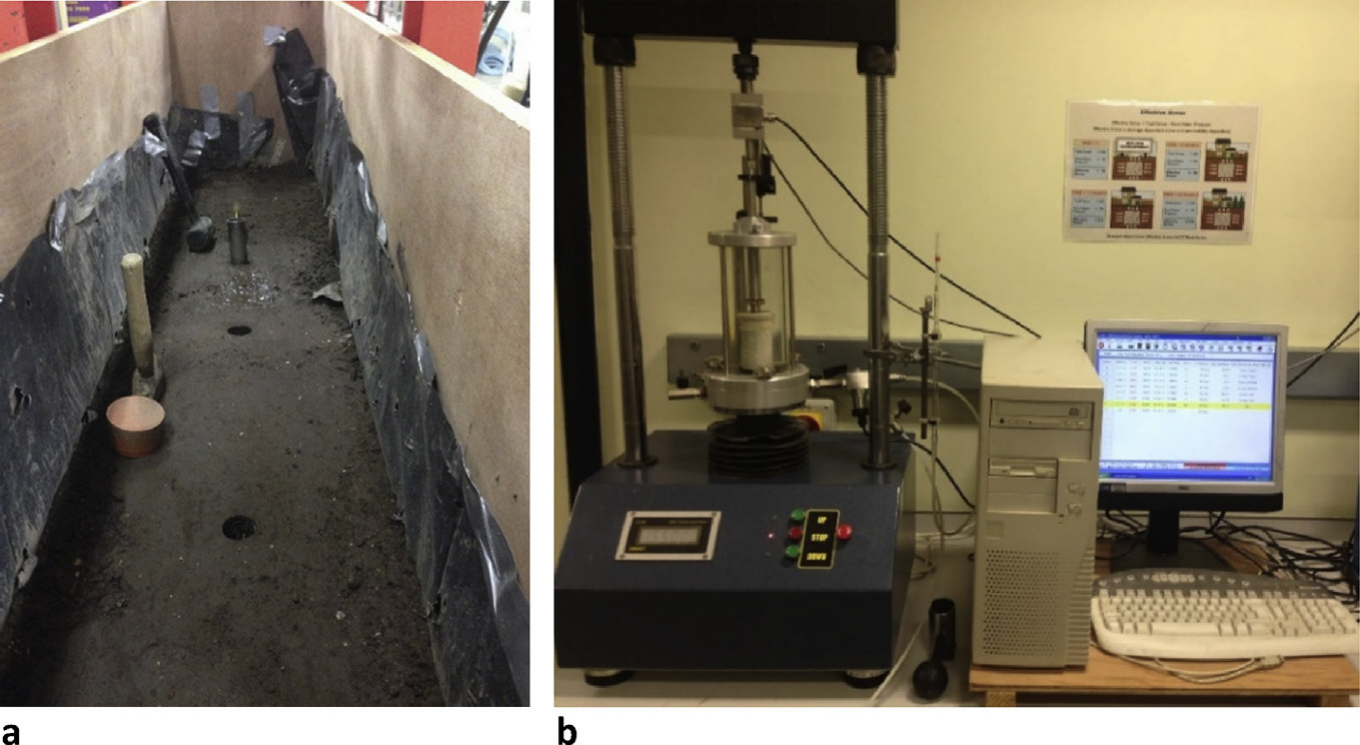
**a.** Extracting the soil specimens from the trench. **b.** The apparatus for the triaxial test.

**Fig. 6:**
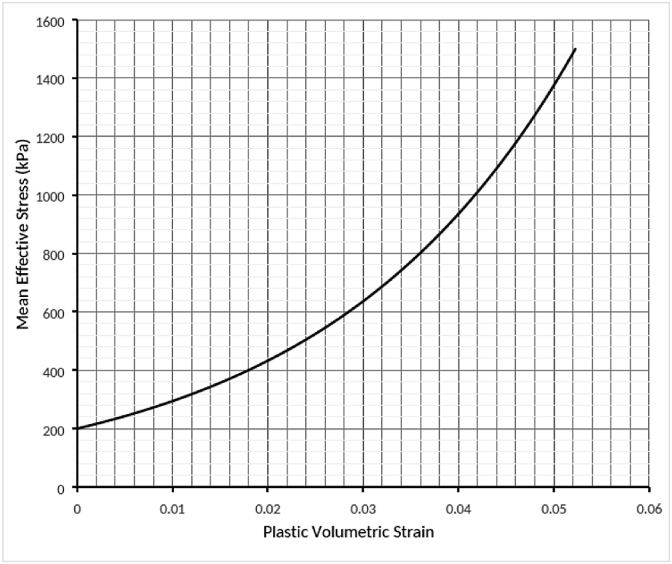
Evaluating the modified cap-hardening curve.

**Fig. 7:**
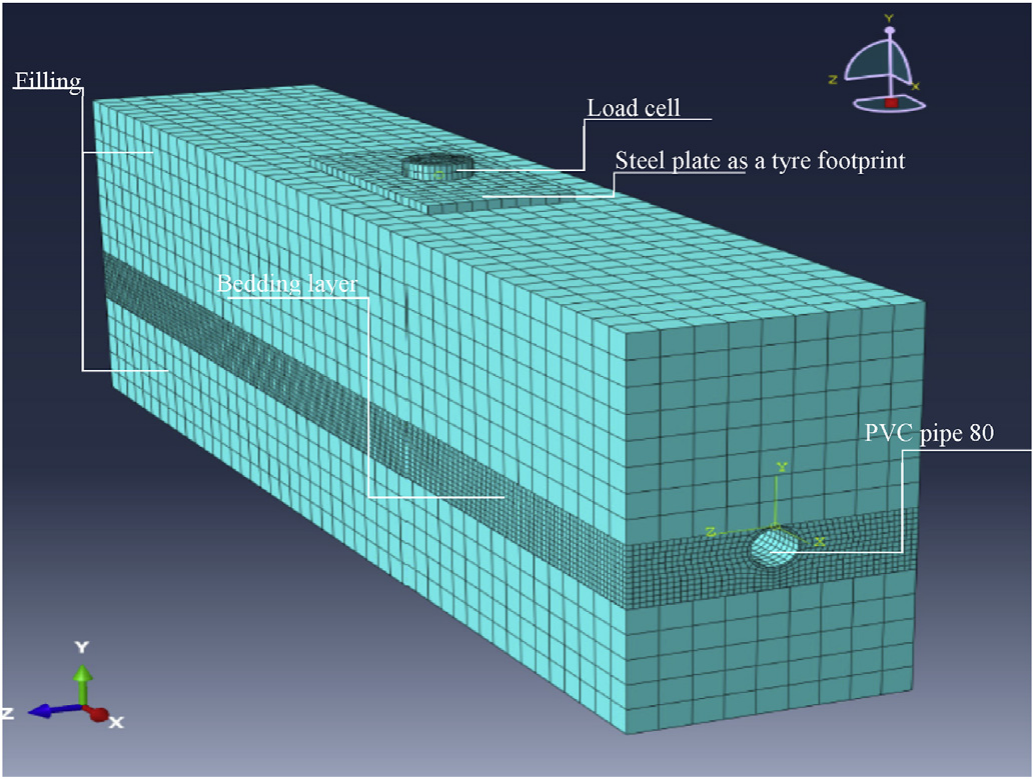
3D FE model used to simulate the physical laboratory model of one pipe set in a trench.

**Fig. 8:**
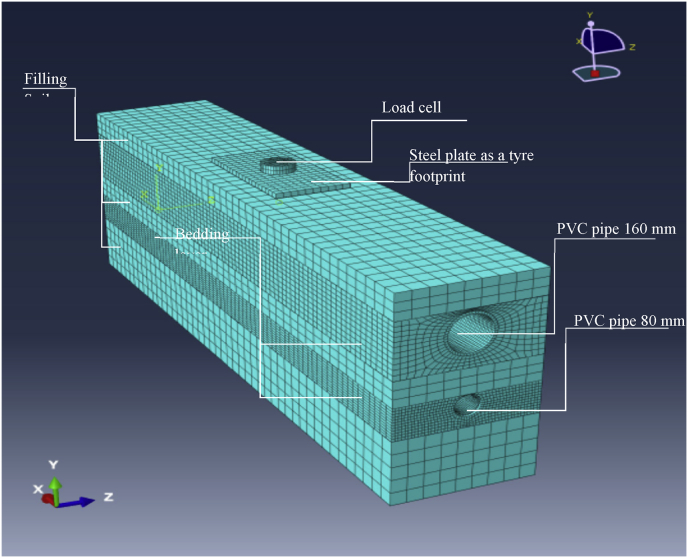
3D FE model used to simulate the physical laboratory model of two pipes set in one trench.

**Fig. 9:**
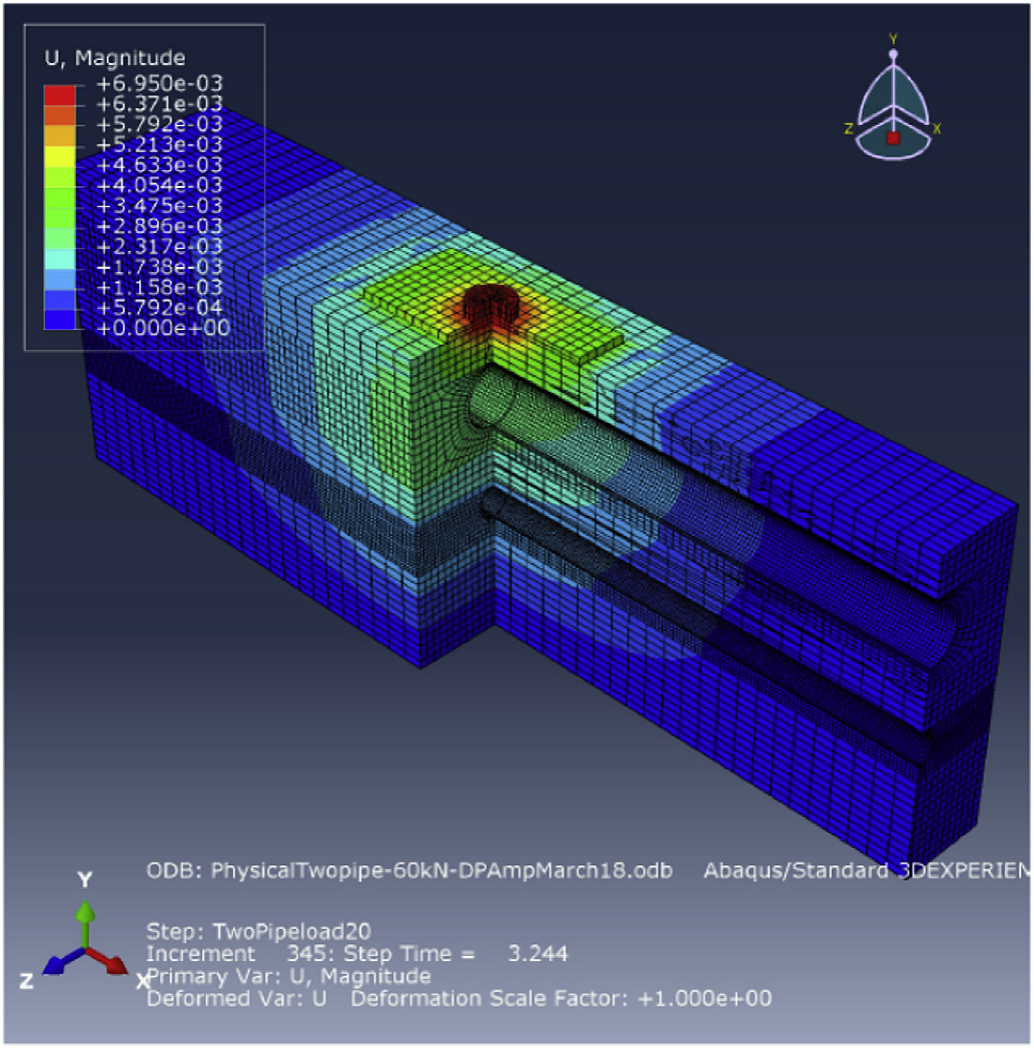
Visualisation results for the FE model with two pipes set in one trench under an H20 traffic load.

**Fig. 10:**
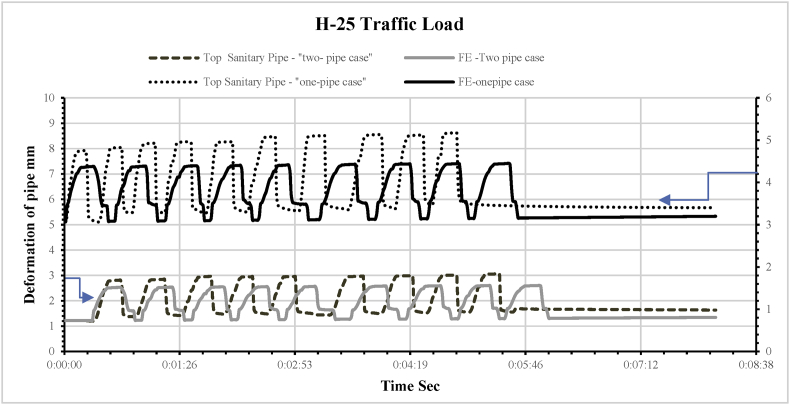
Comparison of the experimental and FE results for the deflection of the small (sanitary) pipe when set alone in the trench and when set with a storm pipe under a traffic load of H25.

**Fig. 11:**
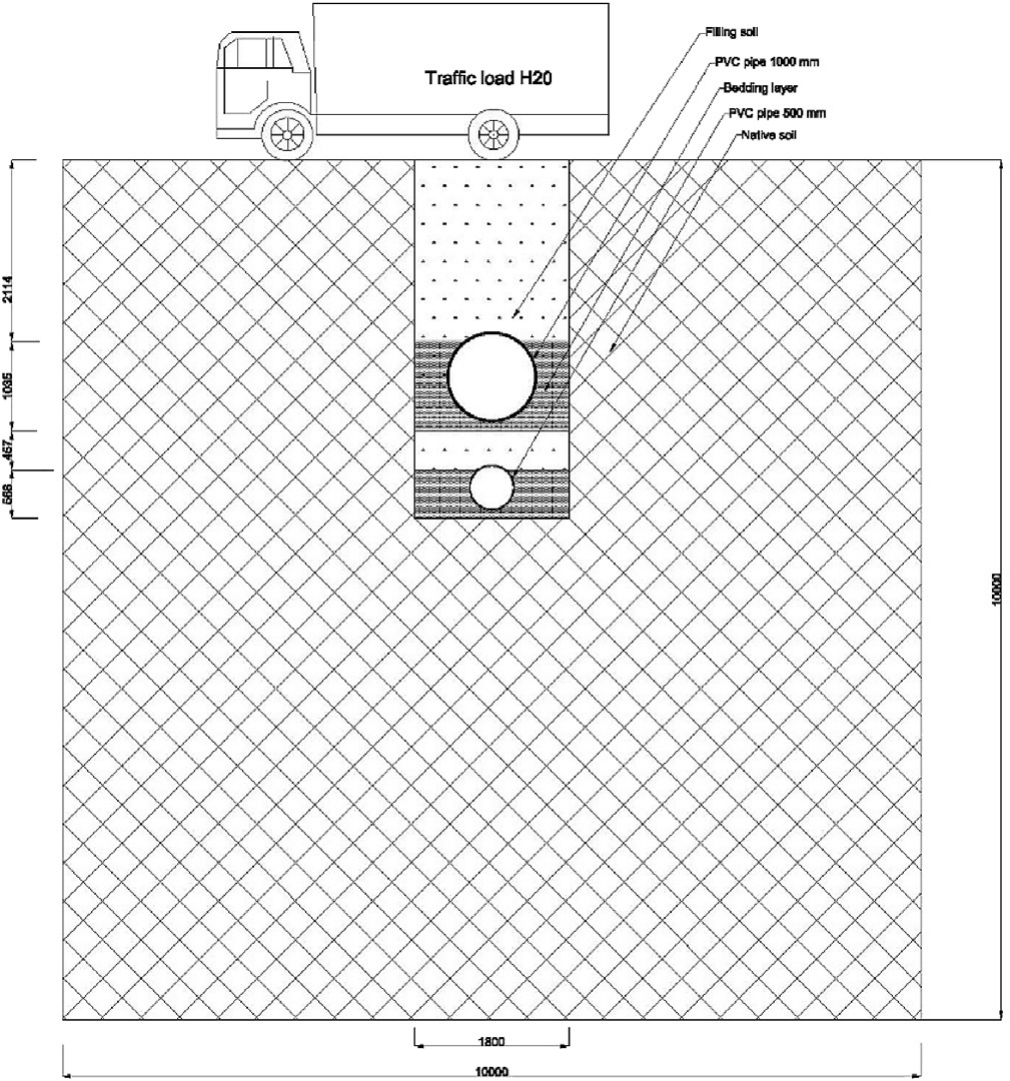
The model of a 1000 mm diameter storm pipe and 500 mm diameter sanitary pipe in one trench.

**Fig. 12:**
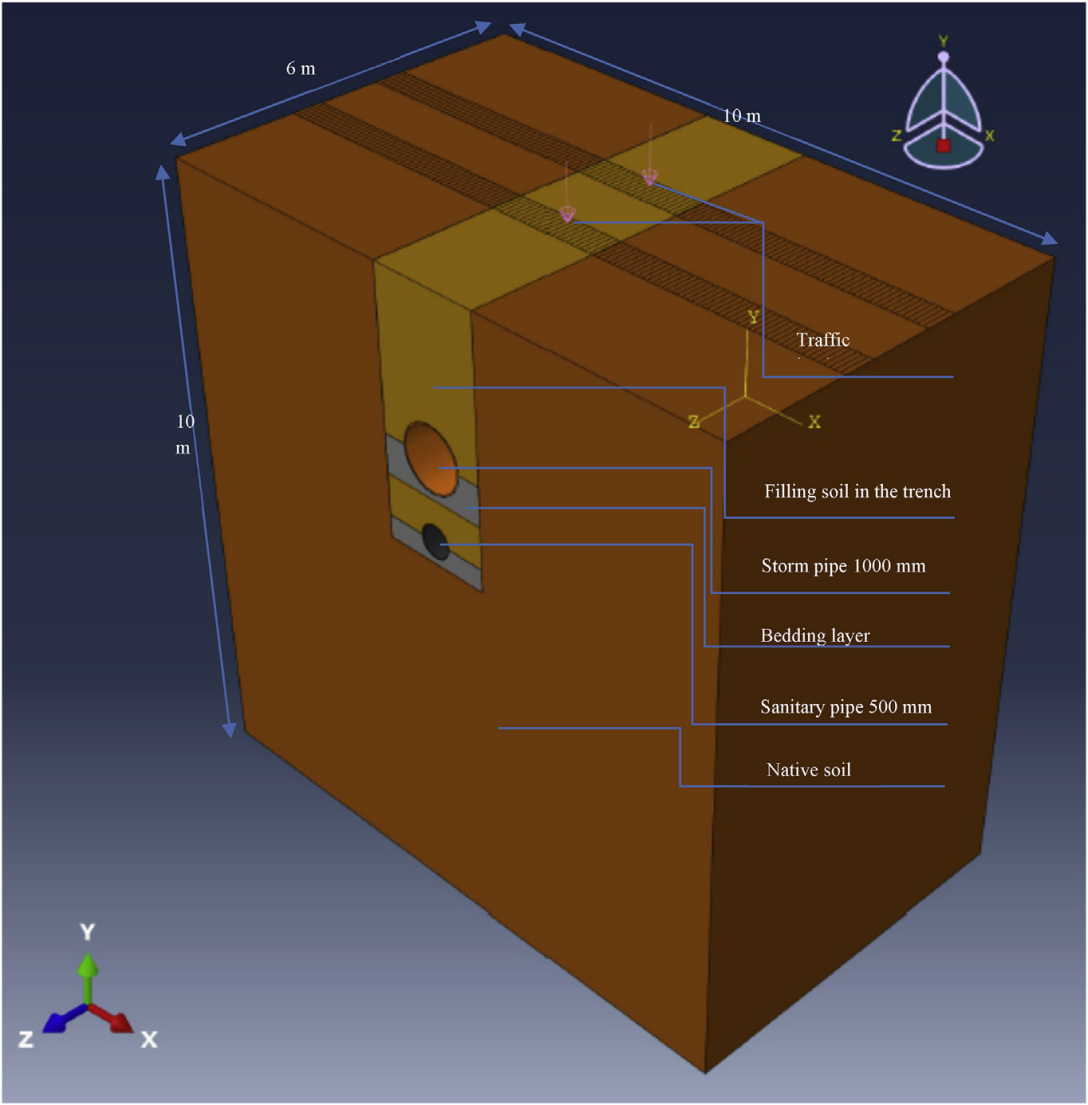
3D FE model of a 1000 mm storm pipe and 500 mm sanitary pipe in one trench.

**Fig. 13:**
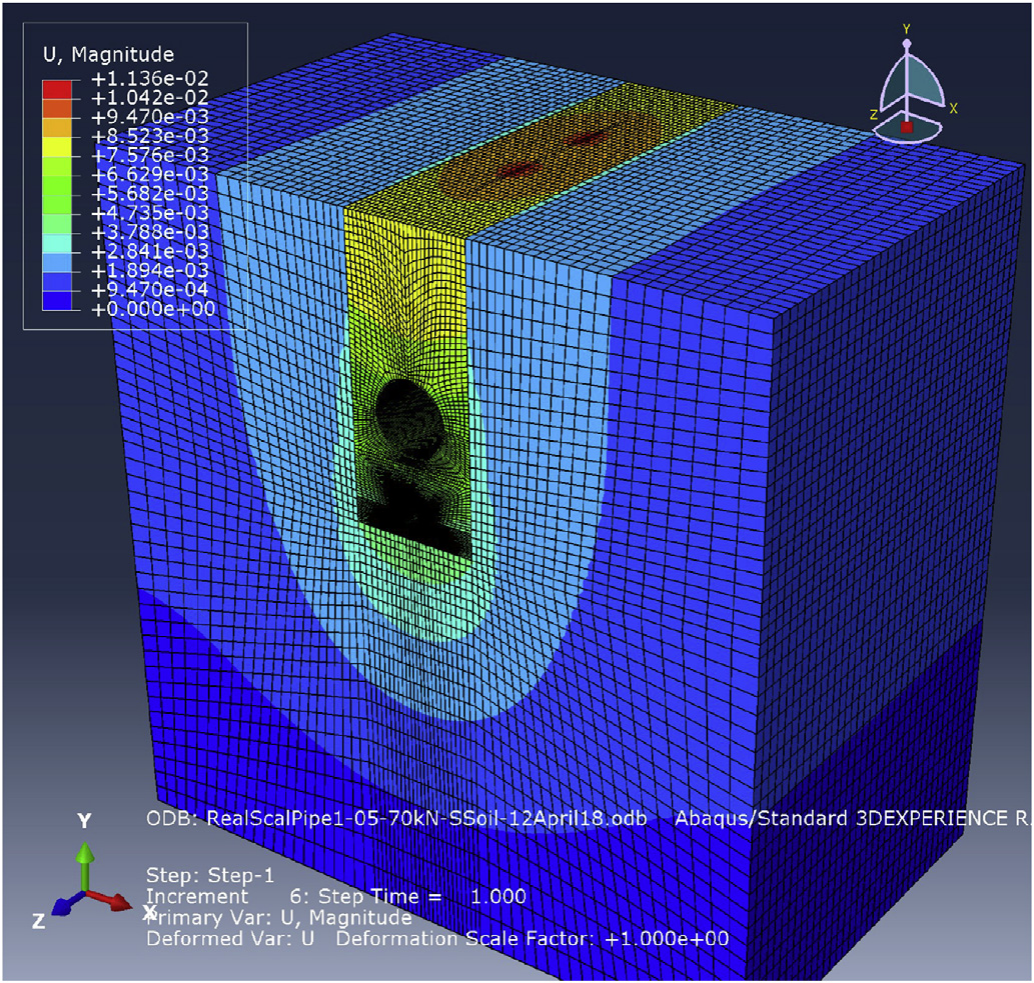
Visualisation results for the FE samples of the real-scale model when two pipes lie in one trench under an applied H20 traffic load.

**Fig. 14:**
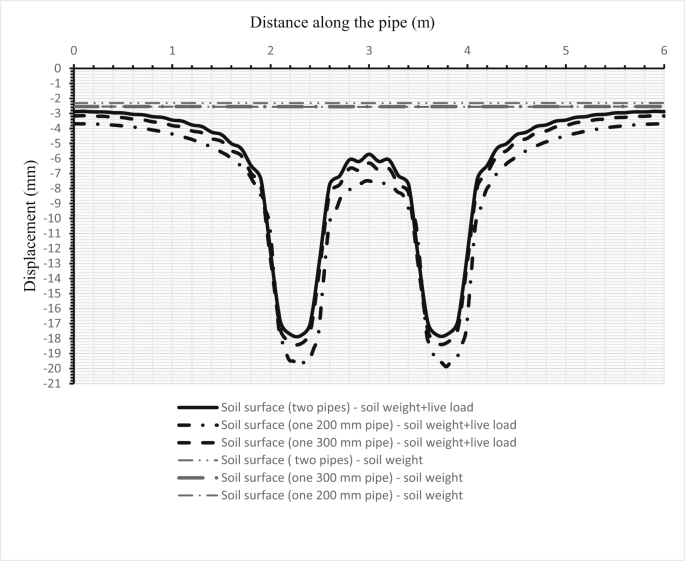
Comparison between the deflections of the soil surface in three cases for two pipes, and when either one sanitary pipe or one storm pipe is set in the trench.

**Fig. 15:**
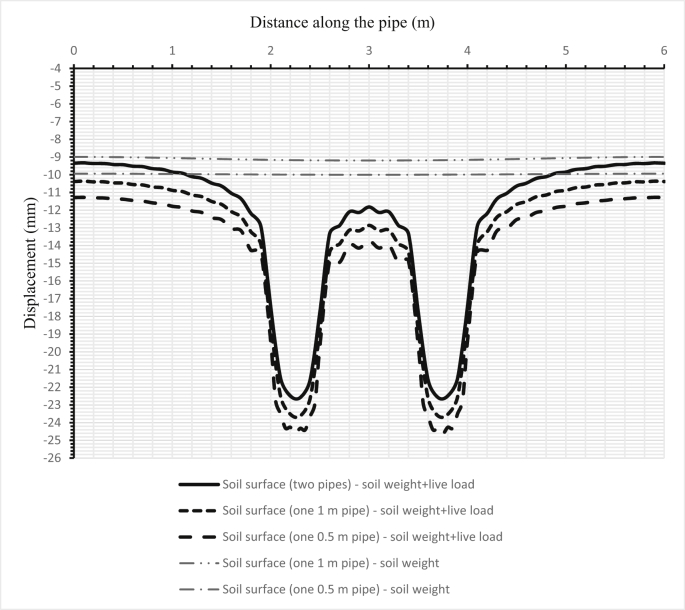
Comparison between the deflections of the soil surface under three cases for two pipes and when either one sanitary pipe or one storm pipe is in the trench for the second set.

**Fig. 16:**
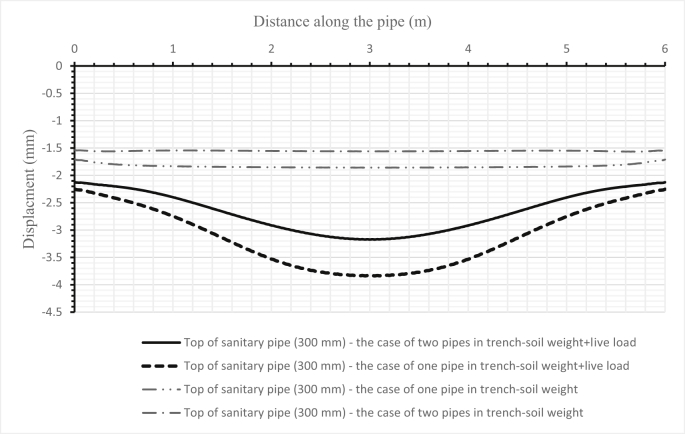
Comparison between the deflections of a sanitary pipe (300 mm) when set alone and when set below a storm pipe (500 mm) in one trench for the first scenario.

**Fig. 17:**
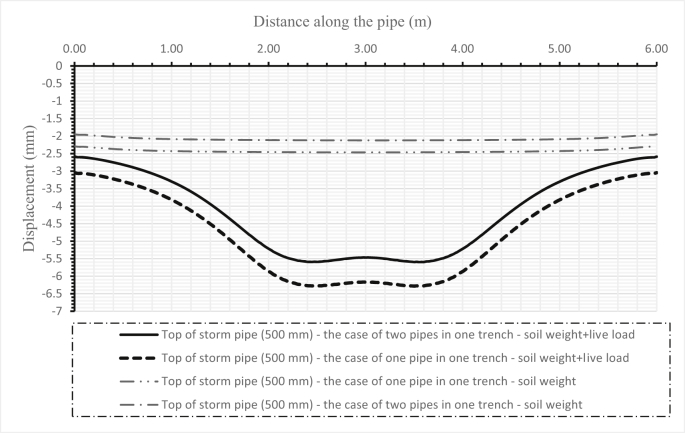
Comparison between the deflections of a storm pipe (500 mm) when set alone and when set above a sanitary pipe (300 mm) in one trench for the first scenario.

**Fig. 18:**
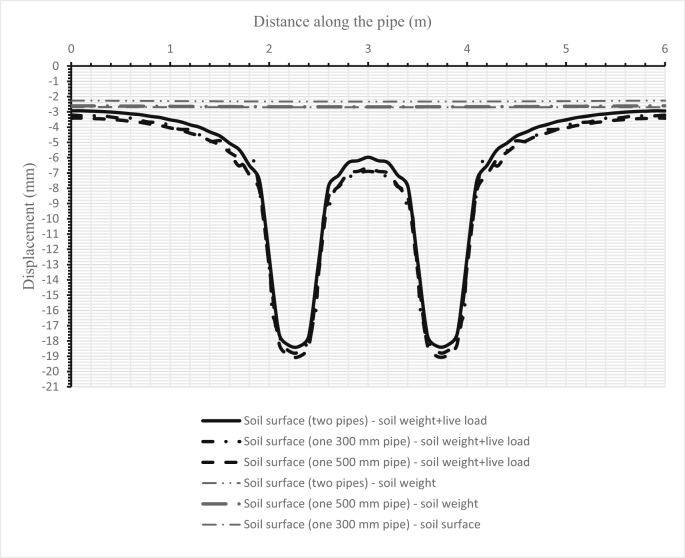
Comparison between the deflections of the soil surface under three cases for two pipes and when either one sanitary pipe or one storm pipe is in the trench for the first scenario.

**Fig. 19:**
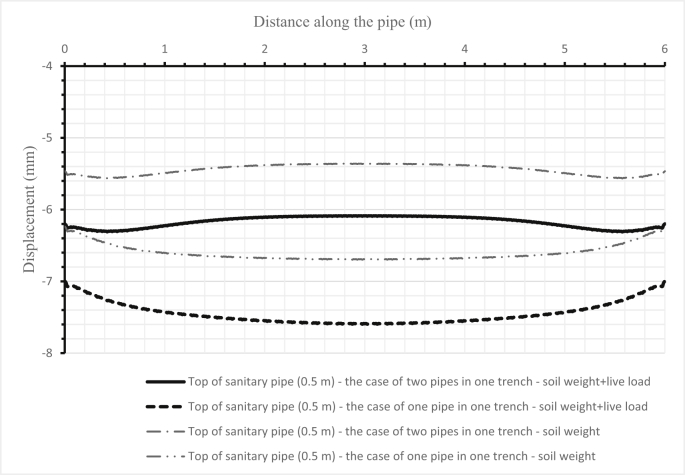
Comparison between the deflections of a sanitary pipe (500 mm) when set alone and when set below a storm pipe in one trench for the second scenario (4 m cover depth).

**Fig. 20:**
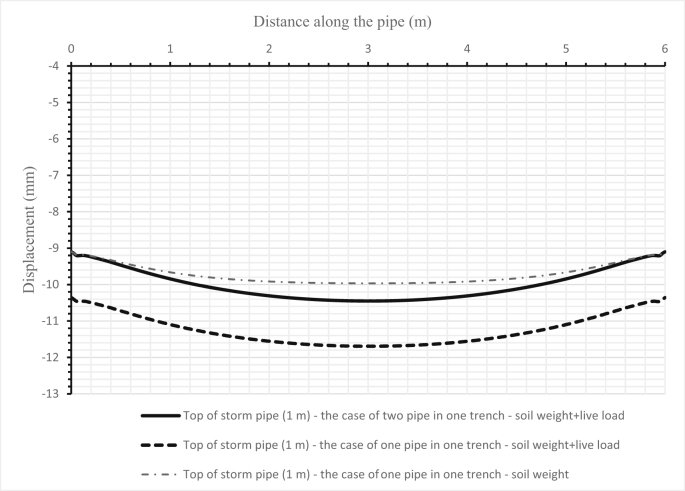
Comparison between the deflections of a storm pipe (1000 mm) when set alone and when set above a sanitary pipe in one trench for the second scenario (4 m cover depth).

**Fig. 21:**
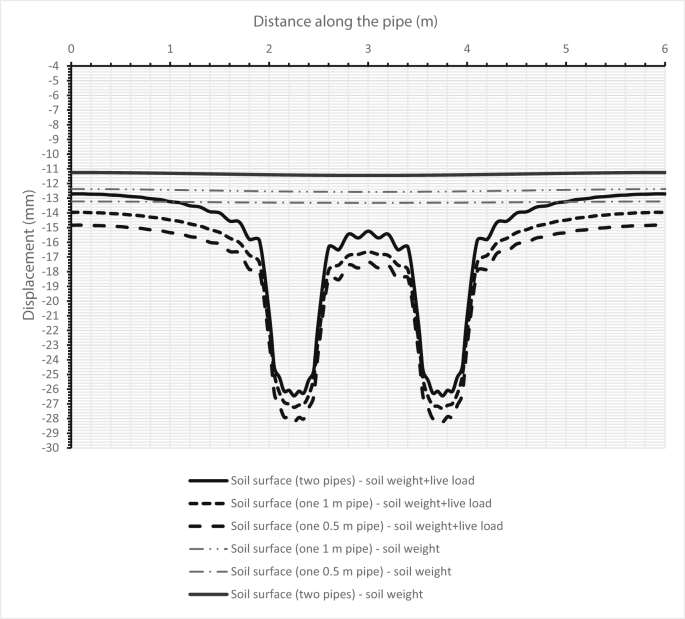
Comparison between the deflections of the soil surface under three cases for two pipes and when either one sanitary pipe or one storm pipe is in the trench for the second scenario (4 m cover depth).

The data presented in this article relates to the structural performance of flexible pipes buried in a trench [1]. This research constitutes a new approach to manhole design by combining the two manholes into a separate system in a one-manhole structure, still keeping both storm flow and sewage flow separate. The new structure has two chambers: an external chamber for stormwater flow and an inner chamber for sewage flow. Fig. 1 details the design of the new manhole. It compares the conventional method of installation of a separate sewer system when one pipe is placed in each trench (Fig. 2), to the new method of installation where two pipes are set in one trench, one on top of the other (Fig. 3). Physical models were used to test the behaviour of the buried pipes in both installations (Fig. 4). The experimental results were then used to validate the FE model of the physical model (Fig. 5, Fig. 6, Fig. 7, Fig. 8, Fig. 9, Fig. 10). The validated FE model was consequently upgraded to a full-scale model to test the structural integrity of the new installation method compared to the traditional method (Fig. 11, Fig. 12, Fig. 13, Fig. 14, Fig. 15, Fig. 16, Fig. 17, Fig. 18, Fig. 19, Fig. 20, Fig. 21).

## 2 Experimental design, materials and methods

There is a lack of field data concerning the configuration of one-over-one pipes installed in one trench; therefore, it was essential to build a physical model in the laboratory to carry out the tests required to identify the mechanical properties and boundary condition parameters for the system under applied loading. As such, a physical model was built in the laboratory to test the performance of two PVC pipes of 80 mm and 160 mm in diameter. The experiential results have been used to validate the FE model.

### 2.1 The physical model

Engineering is basically design and analysis with attention paid to cost, risk and safety. In this section, the design considered is a buried pipe. Analysis is achieved through a model that predicts performance. Mathematical models are convenient while physical, small-scale models are better for complex pipe-soil interaction. The set of principles upon which a model can be related to the prototype for predicting prototype performance is called similitude. Similitude applies to all models— mathematical, small-scale and prototype.

There are three basic steps to achieve similitude:

1. Fundamental variables (FVs) are all the variables that affect the phenomenon. All the FVs must be interdependent.
2. Basic dimensions (BDs) are the dimensions by which the FVs can be written. The basic dimensions for buried pipes are usually force (F) and length (L).
3. Pi terms are combinations of FVs that meet the following three requirements: (a) The number of pi terms must be at least the number of FVs minus the number of BDs. (b) The pi terms must all be dimensionless.

The pi term for the physical model in this research can be written by using:

**Table:** 

FVs	BDs
W = wheel load	F
D = Diameter of pipe	L
EI = wall stiffness	FL
E′ = soil modulus	FL^2^
H = height of soil cover	L
P = all pressures	FL^2^

To calculate the applied load on the physical model, the pi terms P/E′ are used. The models have been designed to have equal pi terms for both the physical model and real-scale model.

(P/E′) physical model = (P/E′) Real-scale model

The assumption is that the same soil could be placed and compacted in the same way for both models. Therefore, all pressures P must be the same in the physical model and at corresponding points in the real-scale model.

A wooden trench, configured in a hydraulic steel rig, was used to lay the two PVC pipes with the large pipe at the top and the small pipe at the bottom (Fig. 4 a and b). The physical model had dimensions of 2.5 × 0.5 × 1 m^3^ and was embedded in the hydraulic rig, which was used to provide lateral support for the trench walls and to apply traffic loads.

### 2.2 Soil properties

Filling soil was added in 5–10 cm thick layers to achieve the required compaction degree. The bedding layer was used to nestle the two pipes, a 160 mm diameter PVC pipe representing the storm pipe and an 80 mm pipe used as a sanitary pipe. Triaxial Consolidated-Undrained (CU) tests were conducted on undisturbed soil specimens obtained from the physical models after the soil was compacted in the trench. Fig. 5 a and b shows the location of the soil specimens extracted from the trench from the first layer of the soil underneath the buried pipes.

The results of these tests were used to identify the soil properties (Table 1) and establish the cap-hardening curve that describes the evolution of the soil's plastic volumetric strain (Fig. 6).

**Table 1:** Parameters of the modified Drucker–Prager cap and Mohr-Coulomb constitutive model for the soil and bedding layer.

Items	Parameters	Value
**Soil**	Density	1685 kg/m^3^
	E	16.943 MPa
	ʋ	0.295
	**Drucker–Prager**	
	β	55
	K	0.8
	ψ	15
	λ	0.044
	κ	0.0056
	e_o_	0.48
	**Mohr–Coulomb**	
	ϕ	31.7
	C	50
**Bedding**	Density	1855 kg/m^3^
	E	75 MPa
	ϕ	35
	C	0
	ʋ	0.4

### 2.3 FE model of the physical model

FE models were created to simulate the physical laboratory model, including the plate of the tyre footprint, the load cell, pipes, bedding layers and filling soil. The models have the same dimensions and boundary conditions as the physical model. Fig. 7 shows the FE model of a sanitary pipe lying alone in the trench, while Fig. 8 shows the FE model consisting of a storm pipe lying above the sanitary pipe in the same trench.

The same series of loads applied in the physical model was used in the FE model to explore the behaviours of the pipes and compare the physical and FE model results for validation. Fig. 9 shows a sample of the visualisation results produced by the FE physical model with two pipes in one trench. The results from applying the H25 load are presented in Fig. 10, illustrating the behaviour of the buried pipe when set alone in the trench and when set below the large pipe for both physical model and FE model.

The results show acceptable consistency, R = 0.93 to 0.95. This shows an acceptable validation process, which allowed the researchers to upgrade the FE model to a full-scale model.

### 2.4 Full-scale FE model

Conventional sewer systems typically use minimum diameters of 200 mm for sanitary networks and 300 mm for storm networks. The minimum cover depth used to provide protection for a sewer system network is 1 m for pipes with diameters of 200–1000 mm and 2 m for pipes with diameters of 1000 mm and above [4,5]. The 3D FE model was applied with the real-scale dimensions of two sets of pipe diameters. The first set included two PVC pipes, a 200 mm diameter sanitary pipe and a 300 mm diameter storm pipe, buried at a soil cover depth of 1 m. The second set also included two PVC pipes, but buried at a soil cover depth of 2 m: a 500 mm sanitary pipe and a 1000 mm storm (Fig. 11).

The width and height of the whole model were selected to measure the extent to which a traffic load can affect the native soil around the trench occupied by the pipes [6]. The dimensions of the model were 10 × 6 × 5 m^3^ for the first set of experiments and 10 × 6 × 10 m^3^ for the second set (Fig. 12).

The ABAQUS 2017 package was used to implement the 3D FE model on the LJMU cluster, as the dimensions of the model required powerful, high-performance computing. The first model (200–300 mm) included 452,564 linear hexahedral elements of type C3D8R, while the second model (500–1000 mm) included 397,764 linear hexahedral elements of the same type. The researchers tried to minimise the mesh distortion as much as possible by using a fine mesh of linear, reduced-integration elements (C3D8R) as recommended by the ABAQUS guidelines. The 3D model used in this research meant using hexahedral (brick-shaped) elements wherever possible. They give the best results for the minimum cost (less running time). Complex geometries can be difficult to mesh completely with hexahedrons; therefore, beam and tetrahedral elements may be used in some analyses.

Fig. 13 shows a sample of the visualisation of the 500–1000 mm diameter model with two pipes set in one trench.

Fig. 14 illustrates the deformation of the surface soil for all three cases for the first set (200–300 mm) of pipes. The first case is when only the sanitary pipe (200 mm) is laid in the trench and the second case is when the storm pipe is laid in the trench, while the third case is when both sanitary and storm pipes are installed in the trench, the sanitary pipe over the storm pipe. Fig. 15 shows the surface soil deformation for the same cases for the second set of pipes (500–1000 mm).

#### 2.4.1 Scenarios showing the impact of changing design parameters on the pipes' behaviours in the novel position

The influence of changing design parameters of the new design such as cover depth or pipe diameters has been investigated in two scenarios. The first scenario investigated the system's behaviour when increasing the storm pipe diameter in the first set from 300 mm to 500 mm. The results of the sanitary pipe deflection are illustrated in Fig. 16, the storm pipe deflection is shown in Fig. 17, and the soil deformation is presented in Fig. 18. The second scenario was applied in the second set by increasing the cover depth from 2 m to 4 m. Results of the second scenario are presented in Fig. 19, Fig. 20, Fig. 21.
